# Comparing the mechanical strength of hip spica cast between a conventional and a new method of application

**DOI:** 10.1007/s11832-016-0770-4

**Published:** 2016-09-10

**Authors:** Kwong-Lee Wan, Rukmanikanthan Shanmugam, Kun-Yun Lee, Aik Saw

**Affiliations:** 1 Department of Orthopaedic Surgery, NOCERAL, University of Malaya, 59100 Kuala Lumpur, Federal Territory Malaysia; 2 Department of Social and Preventive Medicine, University of Malaya, Kuala Lumpur, Malaysia

**Keywords:** Cast breakage, Load to failure, Cast stiffness, Developmental dysplasia of the hip (DDH), Plaster of Paris (POP)

## Abstract

**Purpose:**

The current technique of hip spica application is mostly based on a publication by Kumar (J Pediatr Orthop 1(1):97–99, 1981). We modified the technique of hip spica application in order to reduce the rate of breakage across the hip joint and designed this study to compare the strength between hip spica applied according to Kumar’s technique and the new technique.

**Methods:**

We created 12 hip spica casts with 24 hips according to Kumar’s technique, and another 12 casts according to a new technique. The two types of spica were tested with a mechanical testing machine (Instron 3365 series) by using compression loading to failure in flexion, extension, abduction and adduction. Data were analysed in SPSS version 20.0. Comparison of means was done with an independent *T* test for normally distributed data and the Mann–Whitney test for skewed data.

**Results:**

The new technique involved the creation of three slabs, instead of 14 slabs as described by Kumar. The loads to failure for hip spica cast applied according to the new technique were higher than those applied according to Kumar’s technique, and the differences were statistically significant. The stiffness was also higher in spica casts applied with the new technique; the difference was not statistically significant under extension force.

**Conclusion:**

Hip spica applied with the new technique was stronger than that applied according to Kumar’s technique based on load to failure testing. The new technique of application would potentially reduce the risk of cast breakage during the management of developmental dysplasia of the hip (DDH) and femur fracture in children.

## Introduction

Hip spica is one of the main treatment modalities in paediatric developmental dysplasia of the hip (DDH). In 1981, Kumar described a technique of hip spica cast application [1]. He used multiple slabs for the trunk, hips and legs, as they contributed to the strength of the hip spica. Over the years, various modifications have been developed, but most surgeons still adopt the original technique of application as described by Kumar. Plaster of Paris (POP) has been and still is widely used as the material for hip spica due to its conformability during application and low cost. Fibreglass material has the advantage of being lighter and water-resistant, but higher cost could be a limiting factor.

Breakage of spica cast before the intended period of application is a relatively common problem in the management of DDH [2, 3]. Various modifications in plastering technique have been described to strengthen the spica cast. Hosalkar et al. reported hip spica failure occurring at the pelvis-femoral junction and remedied the failure by adding a leg-to-leg connecting bar [2]. No failure was reported and patients’ carers had significant satisfaction, as the bar provided a place to carry a child in hip spica. However, this technique would require additional time and cast material to construct the connecting bar. Chaudhry et al. modified the leg-to-leg connecting bar by replacing the bar with a fibreglass bar [4]. The bar was constructed with existing fibreglass material and no pre-casting preparation of the bar was required, which reduced the procedure time. Curtis et al. published a technique of treating paediatric femoral fractures with hip spica modified into a pontoon spica that incorporated a wooden splint at the fractured side of the limb [5]. The intention was to splint the fractured femur with a stable hip spica construct and the authors recommended that it was a strong technique for children up to 10 years old. This technique would require pre-operative preparation of the wooden splint in a suitable dimension, and breakage at the thigh region (site of femur fracture) is not common in the management of DDH.

There were very few publications on the mechanical strength of hip spica. Increasing the amount of cast material used was a logical solution to reduce the risk of breakage, but this involved increasing the weight and overall cost. There has been no reported study comparing the mechanical strengths of various modifications of hip spica models. In order to reduce the risk of breakage at the junction between the trunk and thigh, we modified the placement of slabs from those described in Kumar’s technique. We designed this study to compare the physical properties of hip spica casts using the conventional technique as described by Kumar and the new technique used in our institution.

## Materials and methods

### Body model

**Fig. 1:**
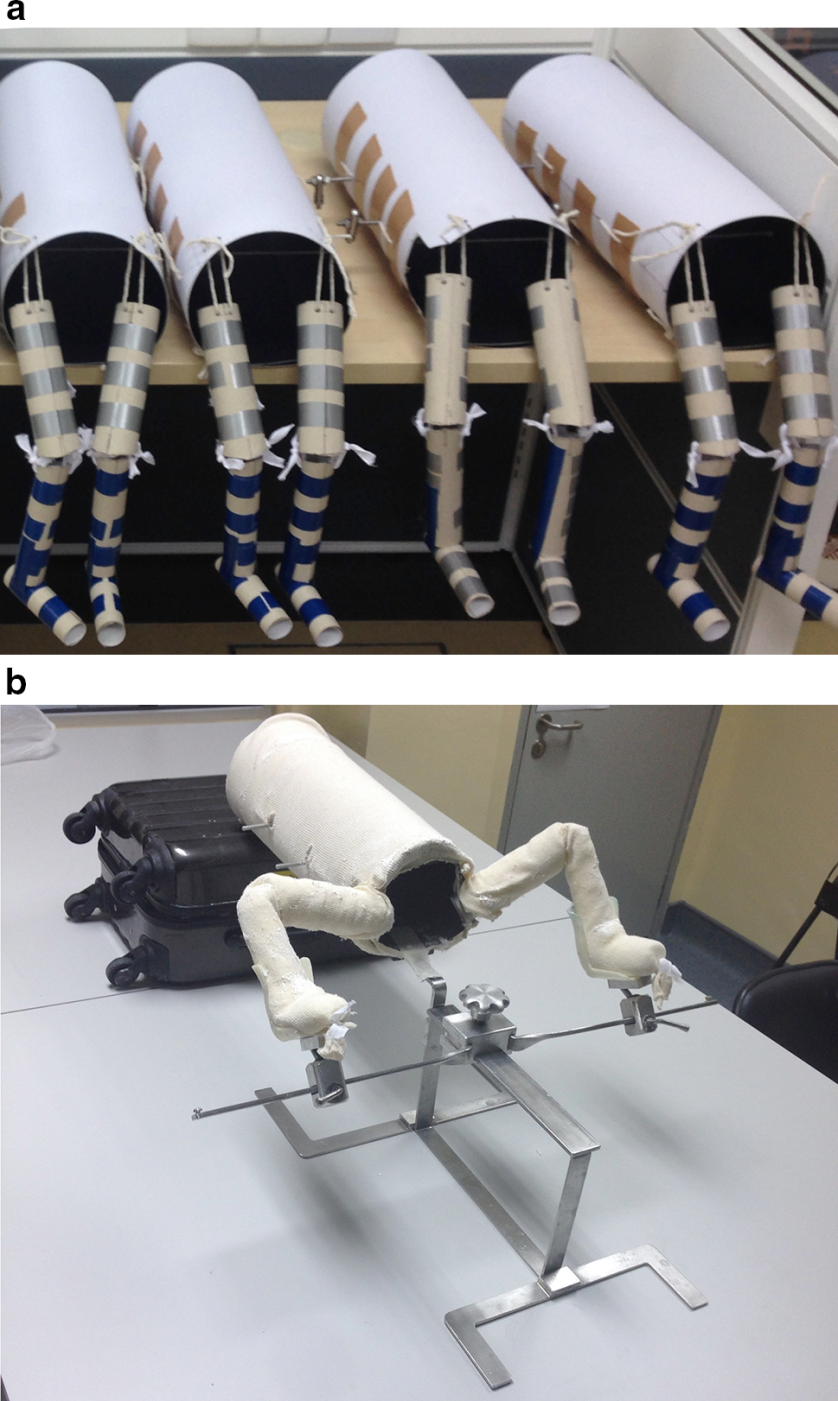
**a** Completed cardboard models of the trunk and limbs. **b** The paper-based model positioned on the self-support frame, ready for plaster of Paris (POP) application. The hip joints could be positioned at 90° flexion and 50° abduction consistently for each hip

**Fig. 2:**
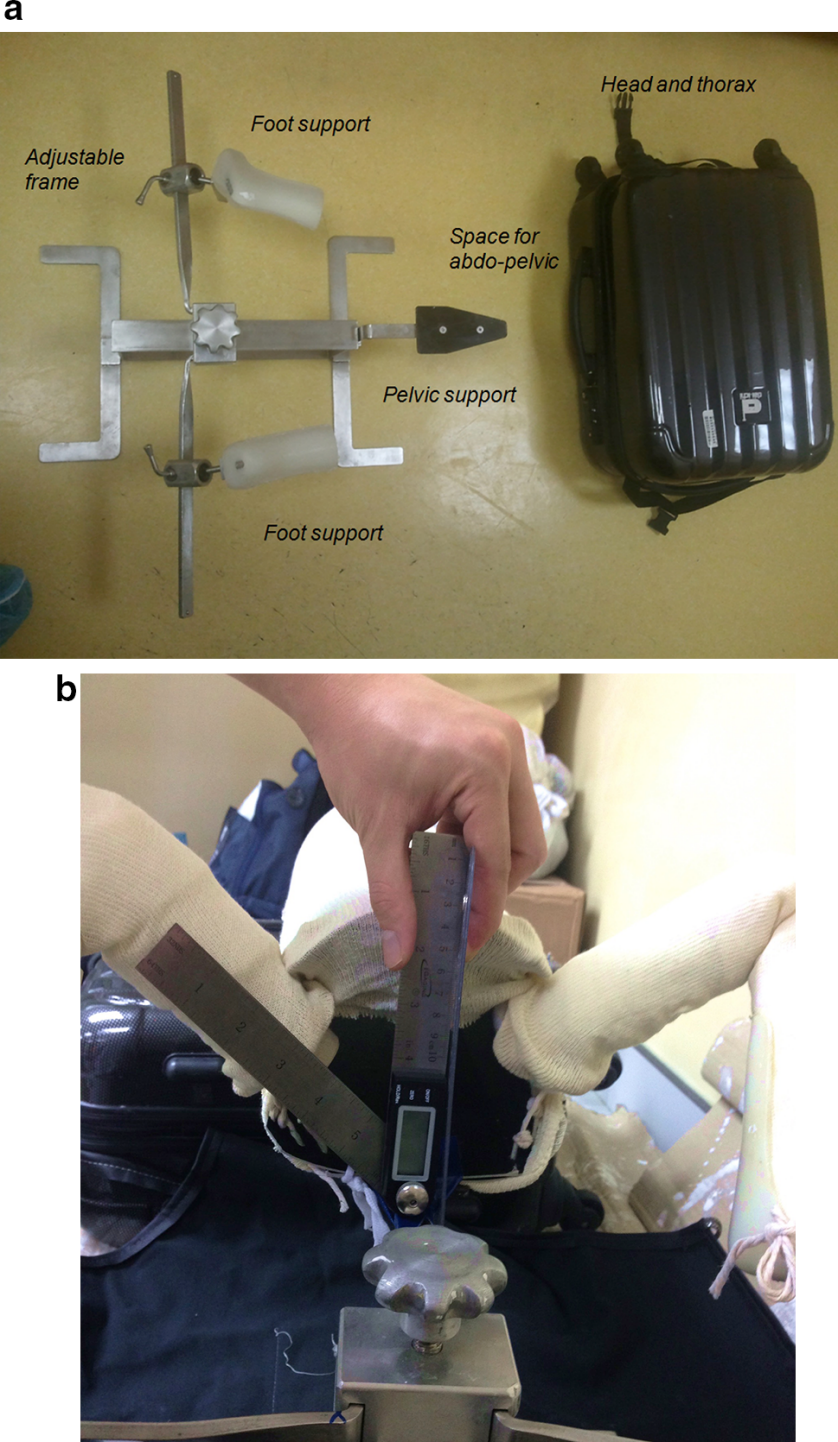
**a** The components of the limb and trunk support frame and its container. **b** Positioning the hip in 50° abduction before application of the cast

The body model was created using cardboard, and it consisted of three components: trunk and two lower limbs. The trunk was made by rolling the cardboard into an oval-shaped hollow cylinder with a vertical inner diameter of 16 cm and transverse inner diameter of 18 cm. The shape was maintained with two threaded metal rods of 6 mm inner diameter applied transversely with free ends protruding from the sides for mounting to the mechanical testing machine. For the limbs, the thigh and leg segments were also made with cardboard rolled into round-shaped hollow cylinders of 4 and 3 cm inner diameters, respectively. Another short segment of hollow cylinder with 3 cm inner diameter was used as the foot segment. We trimmed the ends of the segments obliquely to increase contact for the knee joints. Thick plastic strings were used to connect the components so that they simulated the hip and knee joints during the application of spica cast (Fig. 1a). The legs were wrapped with soft cotton rolls and the whole model was lined with a stockinette (Fig. 1b). This body model was positioned on a frame that we use to support the trunk and lower limbs for applying hip spica cast for small children in clinical practice [6]. The frame allowed the lower limbs to be maintained without the need for an assistant to hold the legs, and this would facilitate the application of hip spica (Fig 2). We positioned the body model with the hip in 90° flexion and 50° abduction to simulate the position for DDH reduction (Fig. 1b). The POP cast material used were 10 cm wide and 2.7 m long per roll (Gypsona® BSN® GmbH, Germany).

### Methods of hip spica application

**Fig. 3:**
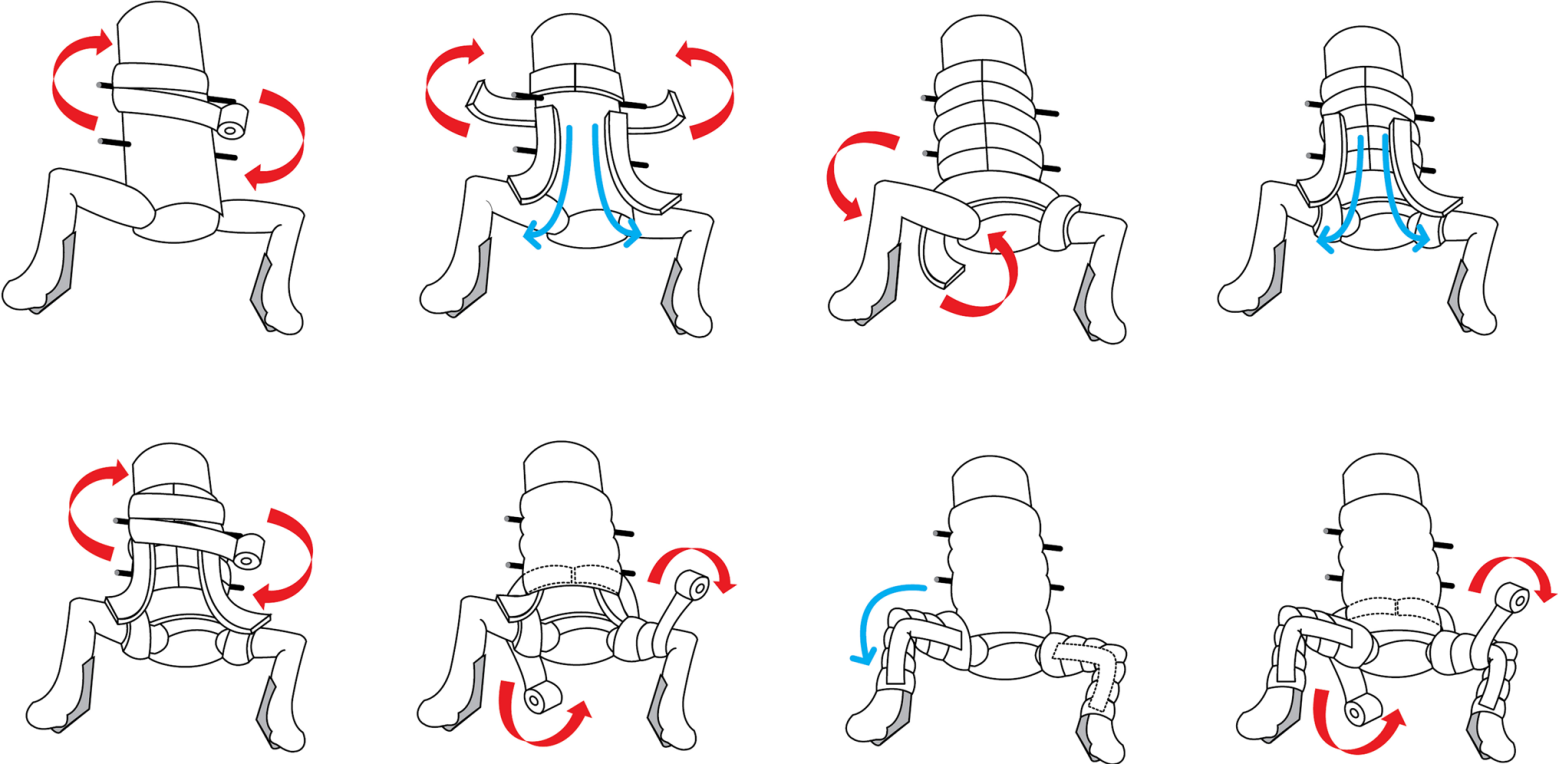
Drawing showing sequential steps of hip spica cast application following Kumar’s technique

For the conventional method of hip spica application, we tried to adhere to the technique as described by Kumar [1]. Since the actual amount of POP material or number of layers used in various slabs was not reported, we used different layers for the slabs (total of 14 slabs), according to the presumed load requirements. We used 16 layers of POP for the transverse anterior slab, eight layers for the slab across the anterolateral aspect of the hip (two sets for both hips, totalling 16 layers) and six layers for the limb slab from thigh to ankle (two sets on each limb, totalling 24 layers). In total, 15 rolls of POP cast were used for each hip spica cast. A step-by-step description of the application is as follows (Fig. 3):

1. First truncal layer.
2. Transverse truncal slabs (five slabs) denoted by the blue arrows. First layer of anterolateral side slab on each side denoted by the green arrows.
3. Transverse truncal-hip slab starting from anterior of the trunk and wrapping around the hip posteriorly and circumferentially.
4. Second layer of anterolateral side slab on each side denoted by the blue arrows.
5. Second truncal layer over the second layer anterolateral side slabs.
6. First layer of hip–leg layer on each side.
7. Lateral and medial side slabs for each leg to strengthen the knee.
8. Second layer of hip–leg layer on each side.

**Fig. 4:**
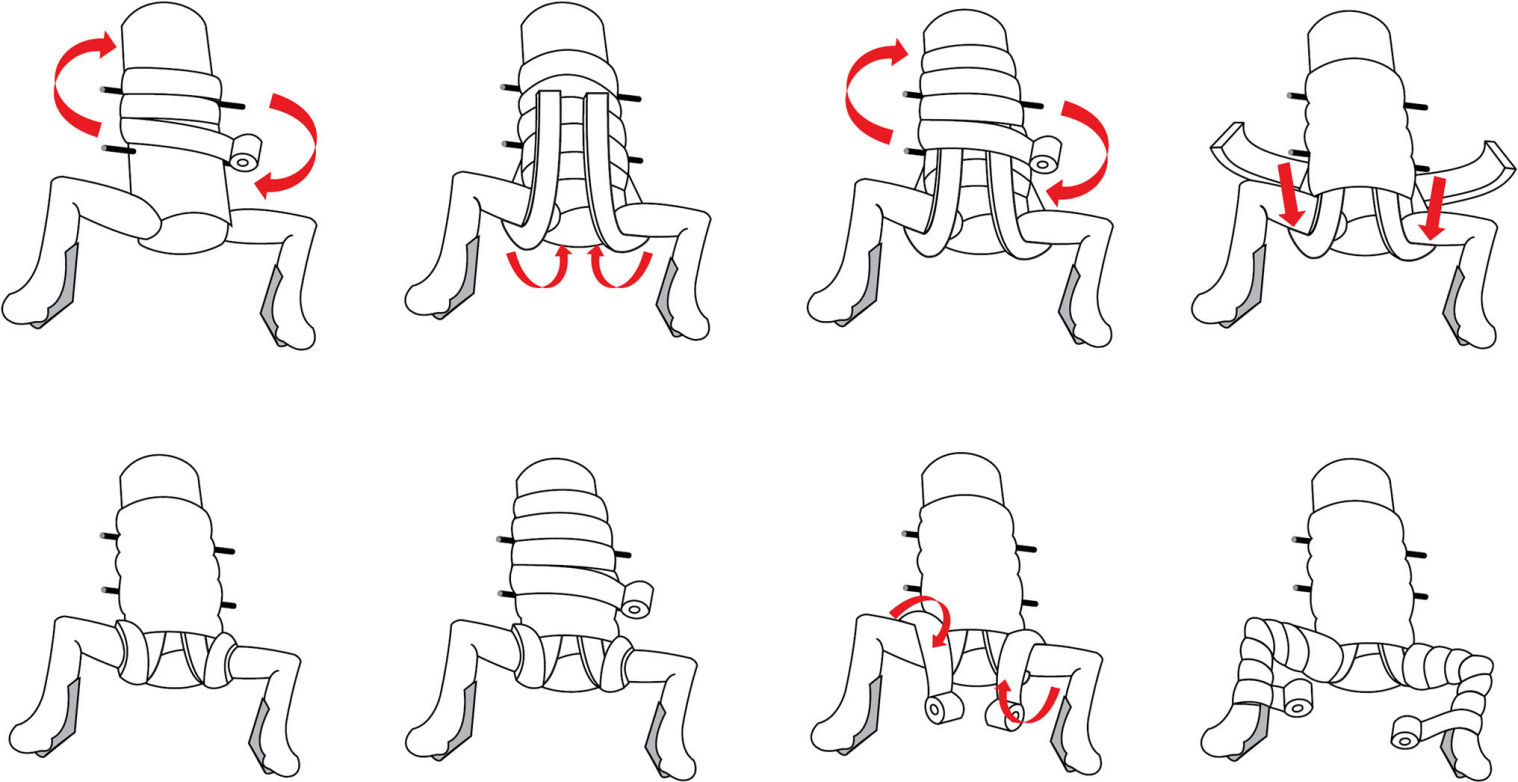
Drawing showing sequential steps of hip spica application according to the new technique

For the new technique, we modified the placement of slabs (total of three slabs) in order to improve the strength of connection between the trunk and limb components. We applied two U-shaped slabs (16 layers of POP material for each slab) across the hips, with the anterior arms tilting towards the midline. Both the arms were overlapped just above the umbilicus region. The posterior arms were placed along the longitudinal axis of the trunk. A transverse slab of 16 layers of POP was placed from the back of the trunk, across the posterior arms to the two U-shaped slaps. The free ends of the transverse slab were brought across the lateral aspect of the hips to be placed over the anterior arms of the U-slabs. The total amount of POP material used for this method was also 15 rolls (Fig. 4). A step-by-step description of the application is as follows:

1. First truncal layer.
2. Two U-shaped slabs applied on each side crossing the hips. Anterior ends would tilt medially and crossed the midline, and posterior ends were parallel to the axis of the trunk.
3. Second truncal layer laid above the U-shaped slabs.
4. Transverse truncal-hip slab applied starting from posterior of the trunk and wrapping anterior of hip joints in circumference. Take note that the slab would be in the opposite position for a similar transverse slab described in Kumar’s technique.
5. Direction of the transverse truncal-hip slab that goes around the hip joint.
6. Third truncal layer.
7. One layer on each hip to the ankle.

A total of 24 hip spica models were created, providing 48 trunk–hip connections for testing. Of these models, 12 were created according to Kumar’s technique and 12 using the new technique. The 24 hip joints in each group were randomised into four groups of flexion, extension, abduction and adduction. Hence, each force direction subgroup had six samples that were tested to failure. All the hip spica casts were created by the principle investigator and dried for 10 days in the same laboratory with a recorded temperature range of 20–27 °C and humidity between 70 and 93 %.

### Mechanical testing

**Fig. 5:**
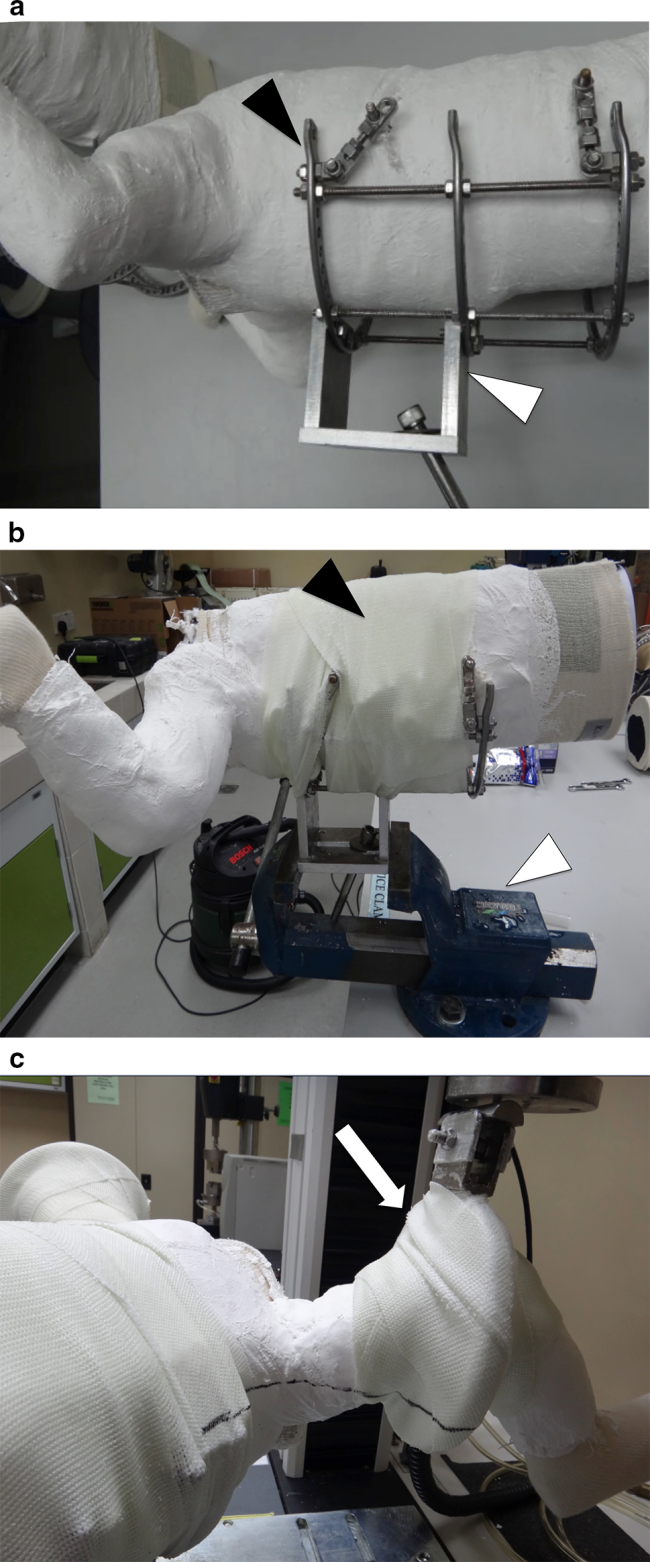
**a** Fixing the holder onto the trunk component with the cast in prone position to measure flexion force. The *white triangle* shows the cuboid connector to the base plate. The *black triangle* shows the Ilizarov external fixator half rings’ connection to the threaded rods crossing the trunk component of the spica cast. **b** Fibreglass cast applied to improve the stability between the holder and trunk element. The *white triangle* shows the vice grip holding the cuboid connector to the table. The *black triangle* shows the fibreglass wrapping the trunk component to the holder. **c** Holder fixed to the lower limb component with fibreglass cast at the level of the knee. The *white arrow* indicates the direction of the loading force

The trunk components of all the spica casts were fixed with 6-mm threaded metal (stainless steel) rods. We created a special holder for the trunk component using three Ilizarov external fixator stainless steel half rings. The holder was fixed to the threaded rods and, in addition, we also used one roll of fibreglass cast to wrap the trunk and holder to reduce potential motion between these components (Fig. 5a). We then mounted the holder to a purpose-built cuboid metal base (Fig. 5b). For the limb component, another holder was created using an Ilizarov external fixator stainless steel full ring. The holder was placed at the level of the knee and secured to the limb POP spica with multiple layers of fibreglass cast material (Fig. 5c). The holder was then connected to a bracket that ensured consistent contact with the load cell of the mechanical testing machine. All applied fibreglass cast material was sprayed with water and left to dry for 24 h prior to testing.

In the flexion and extension test setup, the trunk would be placed in the supine position, with the cuboid metal base attached directly to the baseplate of the mechanical testing machine. In the abduction and adduction test setup, the trunk would be placed in the lateral position, with the cuboid metal base attached to a triangular frame and the triangular frame attached to the baseplate. Additional G- and F-clamps were used to clamp the extended edge of the baseplate to minimise the fixture bending during compression load of the limb component.

The mechanical testing machine used was an Instron® 3365 tabletop universal testing series with Bluehill® software and a 5-kN load cell in compressive mode. The test protocol started with pre-loading the sample at 10 N. The whole test construct was cycled at a rate of 3 mm/s for ten iterations between 0 and 20 N, followed by load to failure at a speed of 1 mm/s. The end of the test was defined as the maximum load of 3000 N or 60 mm vertical displacement.

Data recorded were dry POP weight of each hip spica model, ultimate strength (peak force) and stiffness (force versus displacement) across the trunk–limb connections. The peak force was defined as the first highest force prior to the drop of the force. The stiffness recording was preset at a range of 30–80 N. Data were entered into IBM® SPSS® Statistics version 20.0.0. The distribution of data was tested with the Shapiro–Wilk test. A *p*-value of more than 0.05 was set to assume a normal distribution of data. Normally distributed data comparison was tested with an independent *T* test and a skewed data distribution was tested with the Mann–Whitney test. A *p*-value of less than 0.05 was set to determine a significant comparison difference.

## Results

When we applied flexion force, the means of load to failure for spica casts created using the new technique versus Kumar’s technique were 721.9 versus 371.7 N (*p* < 0.001). When we applied extension force, the means of load to failure for the two hip spica types were 1047 versus 310.3 N (*p* < 0.001). For abduction force, the means of load to failure were 446.3 versus 254.1 N (*p* < 0.001). For adduction force, the values were 439.4 versus 271.4 N (*p* < 0.001) (Table 1).

**Table 1:** Measurements of load to failure and stiffness for four different directions of forces

	New technique	Kumar’s technique	*p*-Value
	Mean ± standard deviation		
Load to failure (Newtons)			
Flexion	721.9 ± 93.6	371.7 ± 60.9	<0.001
Extension	1047.0 ± 112.5	310.3 ± 63.0	<0.001
Abduction	446.3 ± 52.6	254.1 ± 51.9	<0.001
Adduction	439.4 ± 62.2	271.4 ± 21.3	<0.001
Stiffness (Newtons)			
Flexion	243.1 ± 46.1	159.1 ± 35.9	0.006
Extension	282.1 ± 70.0	211.5 ± 35.6	0.052
Abduction	112.8 ± 8.1	58.1 ± 12.8	<0.001
Adduction^a^	117.9 ± 25.4	86.7 ± 17.4	0.026

A *p*-value <0.05 was considered significant

^a^A Mann–Whitney test was used, as data were not normally distributed

We then compared the mean stiffness between the two types of spica cast. Measurements for all the groups showed normal distributions (*p* > 0.05, Shapiro–Wilk test), except for adduction force in spica cast created using Kumar’s technique. Therefore, comparisons of means for flexion, extension and abduction were analysed with independent *T* tests. During flexion, the means of stiffness for the new technique versus Kumar’s technique was 243.1 versus 159.1 N/mm (*p* = 0.006). During extension, the means of stiffness were 282.1 versus 211.5 N/mm (*p* = 0.052). During abduction, the means of stiffness were 112.8 versus 58.1 N/mm (*p* < 0.001). Since adduction data for spica cast using Kumar’s technique were skewed (*p* = 0.034, Shapiro–Wilk test), comparison of means was conducted using non-parametric Mann–Whitney tests. The means of stiffness for the two methods were 117.9 versus 86.7 N/mm (*p* = 0.026) (Table 1).

Based on these analysis, the loads to failure for hip spica created using the new technique were higher than those created using Kumar’s technique by 94, 237, 76 and 62 % in all four force directions of flexion, extension, abduction and adduction. Analysis on stiffness showed that hip spica created using the new technique had higher stiffnesses than those created using Kumar’s technique by 53, 33, 94 and 36 % in terms of flexion, extension, abduction and adduction, respectively, although the difference in extension stiffness was not statistically significant.

The mean dry weight of spica cast created using the new technique was 2.29 kg (±SD 0.06 kg). The mean dry weight of the modified Kumar’s technique group was 2.04 kg (±SD 0.08 kg). The difference in the mean dry weights between the two groups was 0.25 kg, and the difference was significant (*p* < 0.001, independent *T* test).

## Discussion

**Fig. 6:**
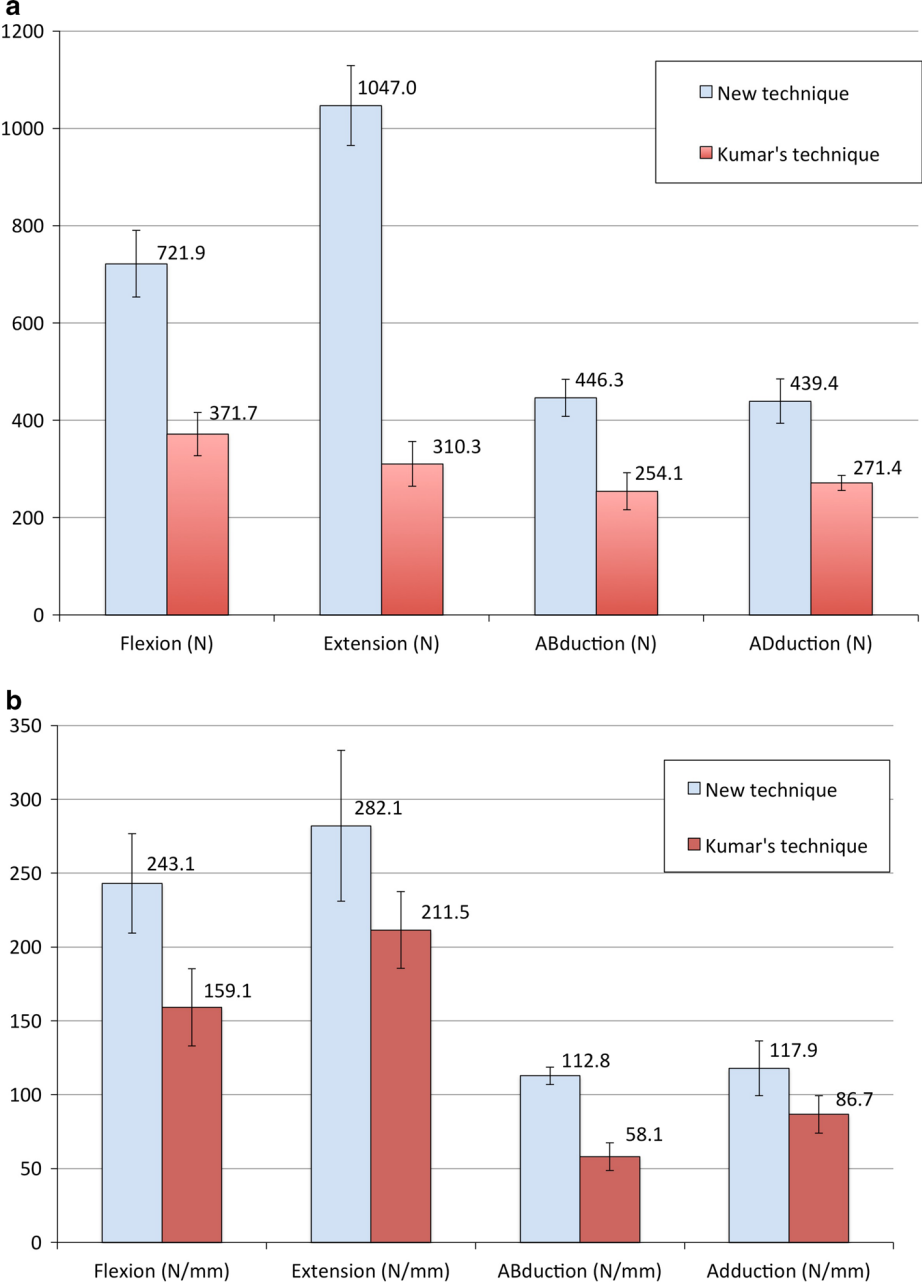
**a** Histogram with error bars shows the means of load to failure under forces of flexion, extension, abduction and adduction between the new technique and the modified Kumar’s technique. The *left bar* (*blue*) is the new technique and the *right bar* (*red*) is Kumar’s technique. **b** Histogram with error bars shows the means of stiffness of flexion, extension, abduction and adduction between the new technique and Kumar’s technique. The *left bar* (*blue*) is the new technique and the *right bar* (*red*) is the modified Kumar’s technique

When we compared the strength between the two types of hip spica, those applied according to the new technique showed higher load to failure in all four forces (flexion, extension, abduction and adduction) compared to those applied according to Kumar’s technique, although the same number of rolls of POP cast material were used (Fig. 6a). Hip spica applied according to the new technique also showed significantly higher stiffness in most of the forces, except for extension force, where the differences were not statistically significant.

With the new technique, the U-slabs extended the anterior and posterior ends of the connection across the hip, increasing the stability against flexion and extension forces (Fig. 4). Moreover, these slabs were actually small plates orientated in the coronal plain, at right angles to the main lateral connection. They provided additional stability in abduction and adduction, similar to the additional stability provided by ridges created for POP slabs across the wrist. Stewart et al. reported that the strength of a volar wrist slab was doubled with the added ridged splint [7]. The ridges were perpendicular to the main volar slab, and they improved stability in the direction of wrist flexion and extension. Another author described moulding the ridged splint following the volar wrist shape [8]. The improvement in stability of the hip spica was supported by higher stiffness and load to failure of our findings, except for stiffness in extension. For Kumar’s technique, the free ends of the anterior slab that were wrapped across the hip would serve to reinforce the posterior ends of the connection (Fig. 3). This may contribute towards higher stiffness against extension force.

For both types of hip spica, the connection between trunk and limb components was able to withstand higher flexion and extension loads compared to abduction and adduction loads (Fig. 6a). POP cast material is strong against compression force, but weak against bending and tension forces. Since the connection across the lateral hip is usually thin (distance between medial and lateral surfaces) and broad (distance between anterior and posterior ends), it is weaker against abduction and abduction forces as compared to flexion and extension forces. These were consistent with our findings (Fig. 6a, b). The anterior end of the connection will withstand the compression force during flexion, while the posterior end will withstand the compression force during extension. During physiological loading, we would expect higher loads in flexion and extension because parents usually try to sit or prop up their child for feeding or playing. This is compatible with the pattern of stability provided by the hip spica. In the new technique, the U-slabs that crossed the front and back of the hip joints would provide more effective stability against flexion and extension forces.

Biomechanical studies on different pelvic osteotomy constructs showed that a maximum deforming force of up to 450 N could be expected following surgery [9, 10]. Our study showed that hip spica created with the new technique would be able to provide adequate support to maintain hip reduction and immobilise pelvic osteotomy in children. However, treatment for DDH in most children may not involve pelvic or femoral osteotomy. Although the angles of immobilisation in pelvic osteotomy may be different and were not tested in this study, based on unreported series of pelvic osteotomies done in our centre, the same casting techniques are able to be used, with no adverse effects so far.

An unexpected outcome of this study was that the mean weight of hip spica applied according to Kumar’s technique was 0.25 kg less than the mean weight of hip spica applied according to the new technique, although equal amounts of POP material (15 rolls) were used. Kumar’s technique required 14 slabs compared to three slabs for the new technique. We postulated that the loss of cast material occurred during the preparation and soaking of slabs. It was possible that less cast material was lost with direct application from the rolls, but we were not able to prove this due to limitations of the study design. Since the number of rolls was the same, the difference in weight may represent an additional advantage of the new technique because more POP cast will be retained during application. Another limitation of the study was that we were only able to compare four deforming forces independently. During physiological loading, the forces usually act together, and not in isolation. We were also not able to test rotation force due to the constraints in the testing design.

One matter that should not be left untouched is the use of fibreglass casts, which is becoming more and more readily available. The use of fibreglass would mean lighter and more durable casts, especially around the perineal area that could get soaked with urine and excrement. Not only is this unhygienic, but it could also weaken the cast around the areas which are critical for support; although we did not test fibreglass casts in this experiment, we expect the outcome to be comparable. If our postulation regarding material loss during soaking is true, then it is possible that using fibreglass material may narrow the gap between the new technique and Kumar’s technique; however, further work is needed in order to examine this aspect.

## Conclusion

Hip spica applied according to the new technique was stronger than that applied according to Kumar’s technique based on load to failure testing. The new technique involved the creation of three slabs, instead of 14 slabs as described by Kumar. The new technique of application would potentially reduce operating time and reduce the risk of hip spica breakage during the management of developmental dysplasia of the hip (DDH) and femur fracture in children.

## References

[1] Kumar SJ (1981). Hip spica application for the treatment of congenital dislocation of the hip. J Pediatr Orthop.

[2] Hosalkar HS, Jones S, Chowdhury M, Chatoo M, Hill RA (2003). Connecting bar for hip spica reinforcement: does it help?. J Pediatr Orthop B.

[3] Ruhullah M, Singh HR, Shah S, Shrestha D (2014). Hip spica versus Rush pins for management of femoral diaphyseal fractures in children. Indian J Orthop.

[4] Chaudhry S, Kang K, Lee MC (2015). Reinforcing a spica cast with a fiberglass bar. Am J Orthop (Belle Mead NJ).

[5] Curtis JF, Killian JT, Alonso JE (1995). Improved treatment of femoral shaft fractures in children utilizing the pontoon spica cast: a long-term follow-up. J Pediatr Orthop.

[6] Chua YP, Saw A, Gunalan R, Kanthan SR (2012). Application of hip spica cast in paediatric. Malays Orthop J.

[7] Stewart T, Cheong W, Barr V, Tang D (2009). Strong and light plaster casts?. Injury.

[8] Chow J, Hsu S, Kwok D, Reagh J (2013). Application techniques for plaster of Paris back slab, resting splint, and thumb spica using ridged reinforcement. J Emerg Nurs.

[9] Adamczyk MJ, Odell T, Oka R, Mahar AT, Pring ME, Lalonde FD, Wenger DR (2007). Biomechanical stability of bioabsorbable screws for fixation of acetabular osteotomies. J Pediatr Orthop.

[10] Yassir W, Mahar A, Aminian A, Newton P, Wenger D (2005). A comparison of the fixation stability of multiple screw constructs for two types of pelvic osteotomies. J Pediatr Orthop.

